# Modeling budbreak precocity in grapevine: insights from comparative gene expression analysis in single-node cuttings

**DOI:** 10.1007/s00425-025-04677-2

**Published:** 2025-04-05

**Authors:** Valeria De Rosa, Emanuele De Paoli, Alessio Angeli, Vittoria Ganzini, Giannina Vizzotto, Rachele Falchi

**Affiliations:** Department of Agricultural, Food, Environmental, and Animal Sciences, University of Udine, Via Delle Scienze 206, 33100 Udine, Italy

**Keywords:** Phenotyping, *Vitis vinifera*, Spring, Budburst, Dormancy

## Abstract

**Main conclusion:**

Single-node cuttings are an effective tool for the study of grapevine’s budbreak timing and cultivar-specific regulation of shared molecular/physiological processes, ABA and *VviFT* key role.

**Abstract:**

Global warming is known to accelerate buds’ phenological development and increase spring frost damage risk in several areas of the world. All studies in this area involve monitoring this intricate process, which is in the field time-consuming and challenging due to the considerable influence of environmental factors. This work explores the possibility of studying dormancy in grapevine by means of single-node cuttings of early- and late-bud break model cultivars Chardonnay and Cabernet Sauvignon. Both visual phenotyping and differential thermal analysis confirmed the expected different pace of dormancy release in the two varieties. In addition, specific Gene Ontology (GO) categories with similar but shifted expression patterns between early-bud break Chardonnay and late-budbreak Cabernet Sauvignon have been identified, suggesting cultivar-specific regulation of shared molecular processes. Notably, the *VviFT* gene aligns with this timing shift, indicating its potential role in budbreak. We further confirm the importance of ABA inhibition in growth resumption and identify genes like *VviSVP2* and *VviDRM1* as possible dormancy release repressors. Our study enhances the understanding of the molecular network underpinning dormancy in grapevine buds and provides a robust framework for future research in this area.

**Supplementary Information:**

The online version contains supplementary material available at 10.1007/s00425-025-04677-2.

## Introduction

The consequences of the changing climate (IPCC 2023) on grapevine cultivation are multiple and diverse, including abiotic stressors, ranging from drought to extreme heat exposure (van Leeuwen and Darriet 2016). The challenge posed by temperature is dual. On one hand, its global increase accelerates plant development (Cameron et al. 2022), prompting early deacclimation and premature loss of cold hardiness in dormant buds. On the other hand, this makes buds susceptible to the risk of late frost damage in spring (De Rosa et al. 2024). Despite the relevance of temperature variation on bud dormancy, the regulation of these responses remains poorly understood. Disentangling the molecular mechanisms involved in dormancy regulation is crucial to contrast the challenges posed by climate change and select, or breed, better-suited late-budbreak varieties (De Rosa et al. 2021).

Given the significant impact of several environmental factors, such as temperature, soil and humidity (Signorelli et al. 2022), monitoring a trait such as budbreak in the field can be difficult, as conditions vary from one season to another. In addition, relying on field data necessitates accounting for several months until temperatures are favourable for deacclimation (Lang et al. 1987). Moreover, budburst is considered reached when more than 50% of buds on the plant have opened (Pellegrino et al. 2022), implying multiple time-consuming observations for precise phenotyping. On these premises, avoiding the inherent variability found in the field to increase standardization is a desirable objective in phenotyping phenological progression.

Single-node cuttings are routinely used in bud dormancy studies to test temperature and chemicals effects (Zheng et al. 2015; Rubio and Pérez 2019), as well as in forcing studies for the generation of budbreak models and time-to-event studies (Fila et al. 2012; Camargo Alvarez et al. 2018). Bud cuttings enable an increase in the number of replicates while requiring minimal space compared to whole plants. Single-node cuttings can also be easily subjected to controlled temperature and humidity conditions, improving the standardization and repeatability of environmental parameters in comparison to the field.

A multitude of molecular studies has underlined the presence of a complex regulative mechanism associated with dormancy regulation and its release. Whereas several key processes have been proven to be involved in these events (Liu and Sherif 2019; Pérez et al. 2021), the knowledge around individual players remains limited (De Rosa et al. 2021). Abscisic acid (ABA) metabolism is one of the most well characterized factors associated with dormancy regulation, with its synthesis playing a central role in the repression of bud meristem activity in grapevine (Zheng et al. 2015). Exogenous ABA application was found to enhance dormancy (Zheng et al. 2015). ABA synthesis is carried out by key enzymes known as *NCEDs* (*9-cis Epoxycarotenoid Dioxygenase*) (Sah et al. 2016). Zheng et al., (2015) described the drop of *VviNCED1* transcript levels following the maximum depth of dormancy in grape buds, while *VviNCED4* expression was observed to specifically correlate with its trends (Shangguan et al. 2020). *Dormancy-Associated MADS-box* (DAMs) genes were also strongly connected to dormancy, as pointed out in revealing studies in *Prunus persica ever-growing* (non-dormant mutant) (Bielenberg et al. 2008). Distinct seasonal expression patterns were observed for individual *DAMs* in peach (Li et al. 2009) and more recently in sweet cherry (Wang et al. 2020) and apple (Falavigna et al. 2021), indicating both chilling requirement and photoperiod as important influencing factors. *DAMs* are closely phylogenetically related to *Arabidopsis thaliana* floral repressor *Short Vegetative Phase* (SVP) (Jiménez et al. 2009), whose nomenclature is retained in non-*Rosaceae* plants such as *Vitis vinifera*. A recent study explored the expression of *VviDAM-SVPs* in grapevine buds during para- and endodormancy, highlighting their differential patterns and associating their regulation to *Flowering Locus T* (*FT*) (Vergara et al. 2021), a flowering time control gene found to be inversely expressed to *SVP* in *A. thaliana* (Jeong et al. 2007). Furthermore, *VviFT* has been associated with the activation of cell cycle genes in grapevine buds treated with dormancy-breaking compound hydrogen cyanamide (H_2_CN_2_) (Vergara et al. 2016). This compound, in addition, indirectly determines the downregulation of *DRM1* (*Dormancy Associated Protein 1*), belonging to the DRM1/ARP family and first identified in pea (Stafstrom 2000). *DRM1* expression is inversely correlated to budbreak occurrence in perennial species such as *Actinidia deliciosa* (Wood et al. 2013) and *Agave americana* (Liu et al. 2023), and it is considered a strong dormancy marker.

In this study, single-node cuttings of grapevine cultivars Cabernet Sauvignon and Chardonnay, considered late- and early-budbreak varieties, respectively (Camargo-Alvarez et al. 2020), were subjected to forcing conditions to evaluate the consistency of their budburst timings in laboratory and field. Buds’ changes were monitored by visual observation and deacclimation progression was confirmed by differential thermal analysis (DTA). Additionally, an in-depth gene expression analysis was performed to further characterize the physiological and molecular differences between the two cultivars, providing insights into the molecular mechanisms underlying their distinct budbreak behaviours.

## Materials and methods

### Plant material and phenological development observation

**Fig. 1:**
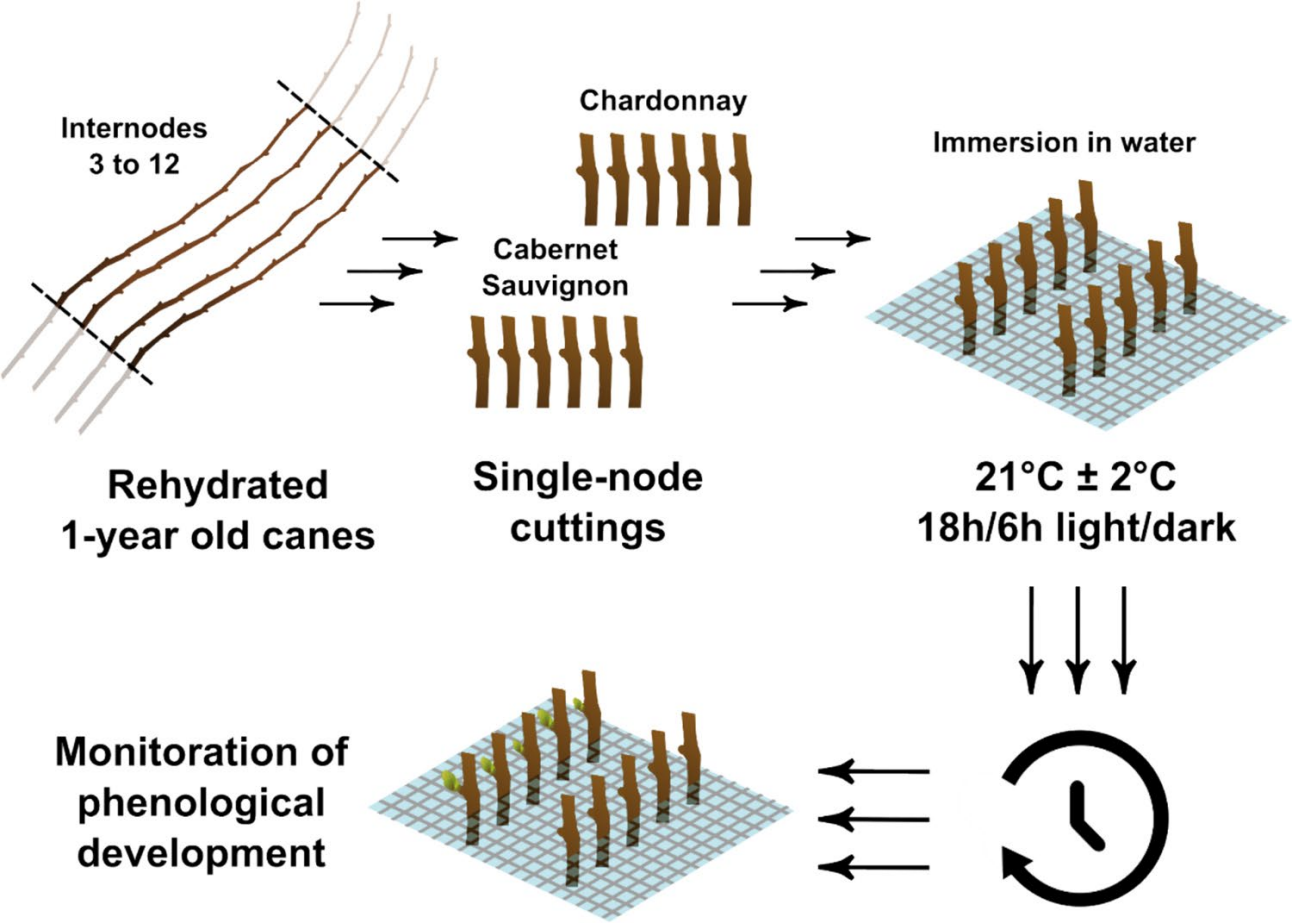
Visual representation of the experimental design

One-year canes of late-budbreak cv. Cabernet Sauvignon and early-budbreak cv. Chardonnay were kindly provided by Vivai Cooperativi Rauscedo (VCR), collected from the field (45°41′N 13°24′E, Grado, Italy) in February 2023 and kept at 4 °C in a dark chamber until the time of analysis. Before starting the experiment, all canes were immersed in water overnight to standardize hydration conditions. Following that, 45 single-node cuttings, from position 3 to position 12 on the cane, were cut and the basal portion was immersed in water at forcing conditions of 21 °C ± 1 °C and 16/8 h of light–dark photoperiod (Fig. 1). For five sampling times, three biological replicates of 5 buds each were regularly sampled for total RNA sequencing and qRT-PCR analysis. Three biological replicates of 5 buds each were simultaneously sampled for differential thermal analysis (DTA). Buds’ phenological development of 20 buds of each variety was monitored visually and classified according to the BBCH scale (Lorenz et al. 1995).

### Differential thermal analysis (DTA)

Cold hardiness determination was performed with DTA using thermoelectric modules (TEM) and temperature probes placed in a T700BXPRO temperature-controlled freezing chamber (FDM, Rome, Italy). Temperature was stabilized at 7 °C for 1 h and subsequently lowered to − 25 °C at a rate of −2.5°Ch^−1^. A CR1000 data-logger (Campbell Scientific, Logan, UT, USA) was used for data recording. Temperature and voltage signals were analysed using RStudio software (R Core Team 2021) using packages *signal* for background noise smoothing (signal developers 2014) and *pracma* for peak analysis (Borchers 2022). Cold hardiness was assimilated to low-temperature exotherms (LTEs), corresponding to peaks of intracellular water freezing events.

### RNA extraction and total RNA sequencing

For each sampling time, excised buds were ground in liquid nitrogen and RNA extraction was performed using the Spectrum™ Plant Total RNA kit (Sigma-Aldrich, St. Louis, MO, USA). RNA quality was checked using NanoDrop™ 1000 Spectrophotometer (Thermo Fisher Scientific, Waltham, MA, USA), and RNA quantity was determined with Qubit™ RNA Broad-Range Assay kit (Thermo Fisher Scientific) using Qubit™ 4 Fluorometer (Thermo Fisher Scientific). Library preparation was performed with TruSeq RNA Library Prep Kit v1 rev. A (Illumina, San Diego, CA, USA) and sequenced on Illumina NovaSeq™ 6000 platform (Illumina) by IGA Technology Services Srl (https://igatechnology.com/). RNA samples were quantified, and quality tested by 2100 Bioanalyzer RNA assay (Agilent Technologies, Santa Clara, CA). Final libraries were checked with both Qubit™ 2.0 Fluorometer (Invitrogen, Carlsbad, CA) and Bioanalyzer DNA assay.

### Read mapping and analysis of Differential Gene Expression

RNA sequencing produced 150 bp paired-end reads. Sequencing adapters and low-quality bases were masked using the Trimmomatic tool (Bolger et al. 2014). Quality check was performed on masked reads with FastQC (Andrews, 2010). Reads were mapped to the telomere-to-telomere (T2T) reference genome PN40024.T2T (Shi et al. 2023) using STAR software package v2.7.9a (Dobin et al. 2013) with default parameters and the v5.1 gene annotation. Read counting per gene was carried out with STAR using the “–quantMode GeneCounts” option, to produce counts coinciding with those produced by htseq-count with default parameters. Differential gene expression analysis was performed with the DESeq2 R package (Love et al. 2014) with default settings. Differentially expressed genes (DEGs) between time points were identified based on |log2FoldChange| ratios > 1 and adjusted p-value (padj) < 0.05. Homoscedastic gene counts, with variance stabilized across the mean, were produced with the variance stabilizing transformation (VST, Anders and Huber 2010) and the regularized logarithm transformation implemented in DESeq2 and applied in the following analyses. Principal Component Analysis was performed using the standard R function *prcomp*() on gene counts normalized by DESeq2.

### k-means clustering and functional annotation

*K*-means clustering was performed to detect prominent gene expression trends among sequenced transcripts, using the *stats* R package with default parameters to group genes with similar expression profiles into *k* clusters based on their normalized expression values. This involved determining the optimal number of clusters *k* using the elbow method, which evaluates the total within-cluster sum of squares (total withinness) for different values of *k* and identifies the point where the marginal gain in reducing within-cluster variance diminishes significantly. Cluster robustness was validated through visualization (boxplot) and clusters exhibiting similar variation profiles across the time course but differing in absolute expression levels were further grouped to simplify the interpretation of global expression trends.

Gene Ontology term enrichment of each cluster was carried out using the topGO R package (Alexa and Rahnenfuhrer 2021) with Fisher’s exact test. Significantly enriched (*p* < 0.001) terms of the ‘Biological Processes’ category were plotted using *enrichplot* R package (Yu 2024).

## qRT-PCR

cDNA was synthesized using QuantiTect® Reverse Transcription kit (Qiagen, Hilden, Germany), and qRT-PCR was carried out with SsoFast™ EvaGreen® Supermix (Bio-Rad, Hercules, CA, USA). Primers used to detect gene expression were designed using the Primer3 tool (https://primer3.ut.ee/) (Table S1) on sequences known in the literature or extracted from the Phytozome database (Goodstein et al. 2012). In detail, the gene sequence for putative grapevine *DRM1* (*Dormancy Associated Protein-Like 1*) was obtained using the full coding sequence of *A. thaliana* homolog AT1G28330 in TAIR10 release as a query for BLAST search within the *V. vinifera* v2.1 genome in Phytozome database. Ubiquitin Conjugating Factor (Vitvi05_01chr19g11590) was used as housekeeping gene for qRT-PCR data normalization (De Rosa et al. 2022).

### Statistical analysis

Statistical significance of DTA and gene expression data was checked using one-way ANOVA and Tukey HSD as post hoc test with SigmaPlot 14.0 software (www.systat.de).

## Results

### Phenological development

**Fig. 2:**
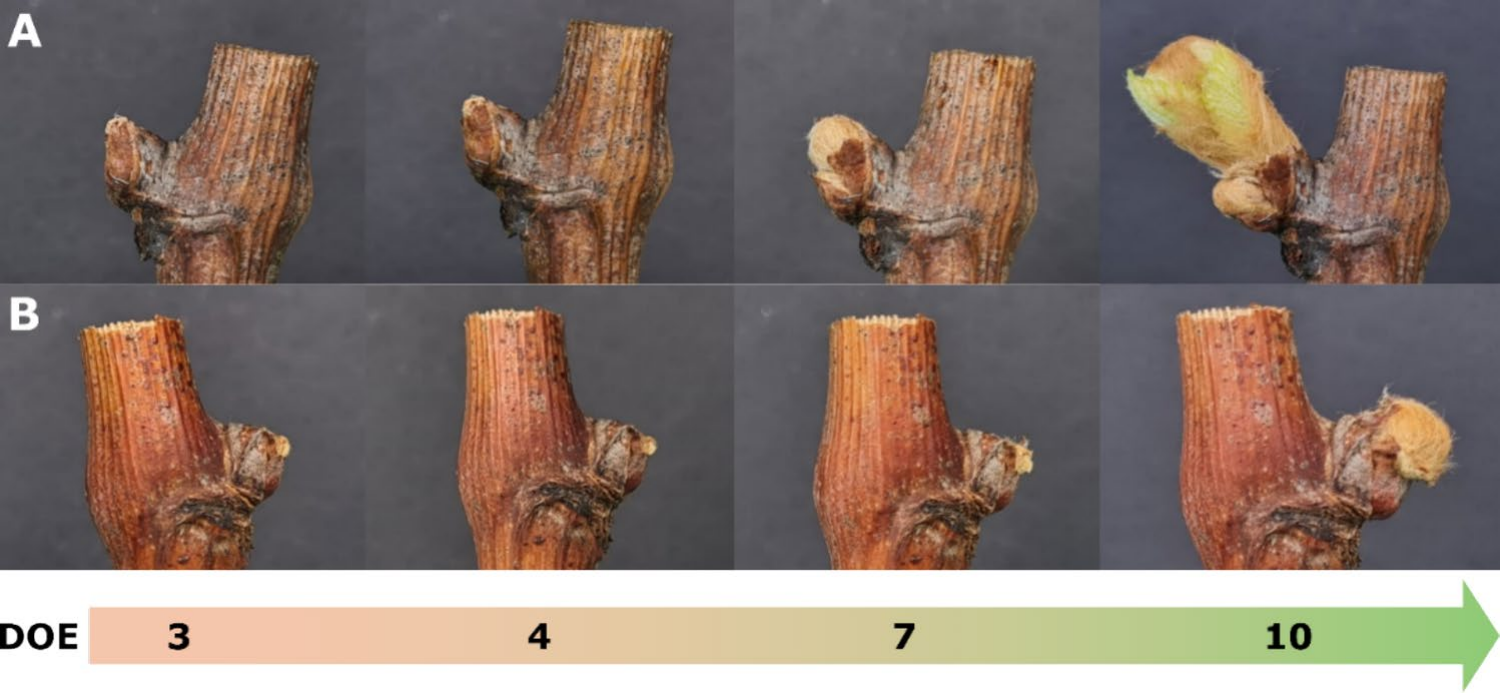
Visual monitoring of phenological development of cvs. Chardonnay (**A**) and Cabernet Sauvignon (**B**) buds throughout the experiment. Chardonnay, in sequence: BBCH 01, BBCH 03, BBCH 05, BBCH 10. Cabernet Sauvignon, in sequence: BBCH 00, BBCH 01, BBCH 02, BBCH 05. Days of the experiment (DOE) are shown on the timeline below

The phenological development of single-node cuttings of varieties Cabernet Sauvignon and Chardonnay showed considerable differences in forcing conditions and was monitored by visual evaluation for ten days (Fig. 2). The classification of the different development stages was carried out based on the general BBCH scale as per Lorenz et al. (1995).

**Fig. 3:**
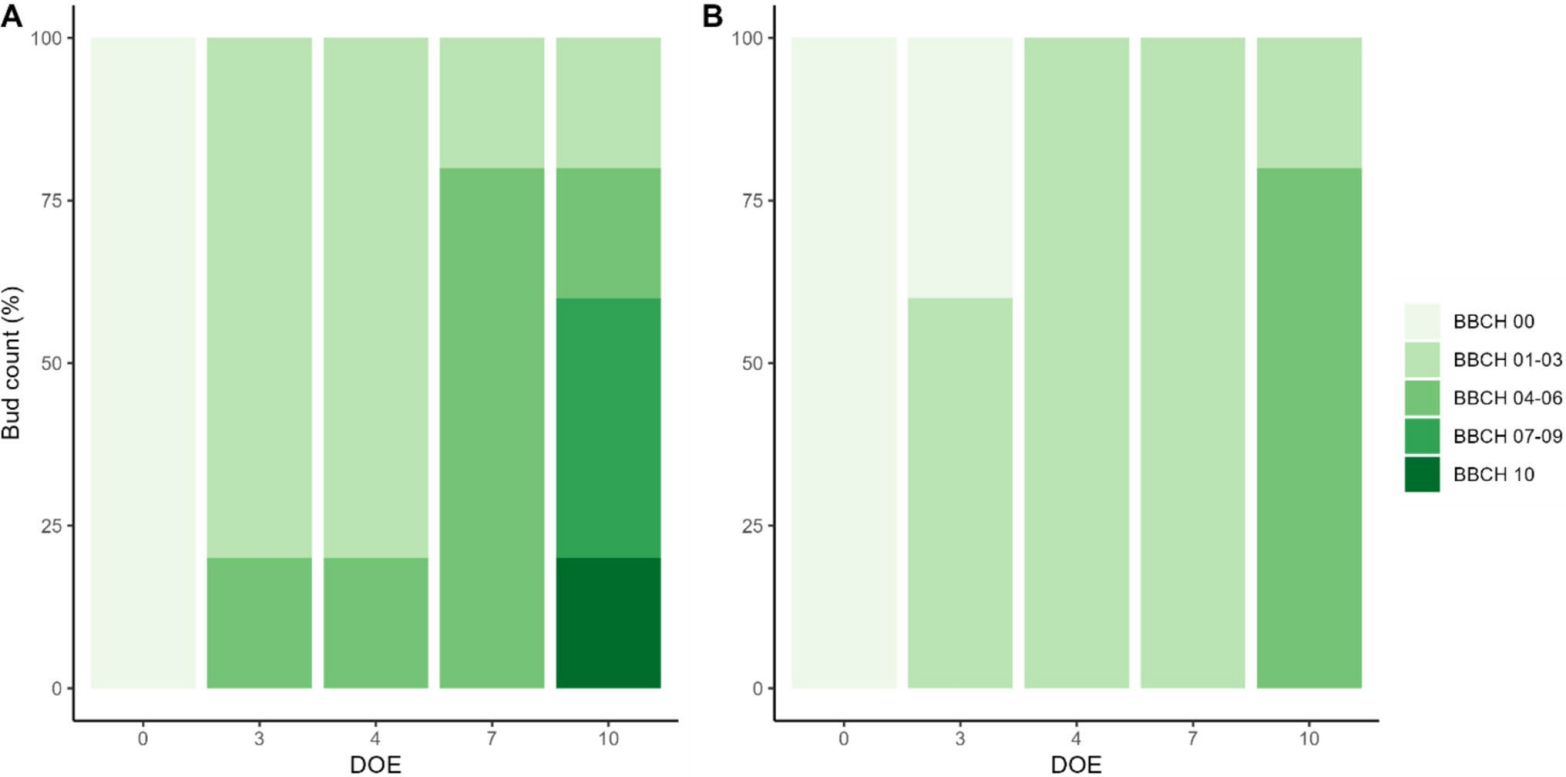
Dynamics of phenological development of buds of cvs. Chardonnay **A** and Cabernet Sauvignon **B** throughout the experiment classified as per BBCH scale. *DOE* day of experiment

In detail, ten BBCH stages, ranging from BBCH 00 (winter bud) to BBCH 10 (first leaf unfolds), were recognized and grouped, allowing a more concise representation of phenotyping (Fig. 3). As expected, early-budbreak cv. Chardonnay appeared generally faster in its progression, showing higher percentages of buds reaching more advanced stages at all sampling times, as compared to the late-budbreak Cabernet Sauvignon. At 3 DOE, all of cv. Chardonnay buds had already entered the swelling phase (BBCH 01–03) with 20% progressing further into the wool stages (BBCH 04–06). Although this condition persisted with no change at 4 DOE, by 7 DOE the percentage of buds at the wool stage had increased to 80%. Then, at 10 DOE, the budburst stage was already visible, with 40% of buds at BBCH 07–09 (bud opening) and 20% of buds reaching the most advanced stage at BBCH 10 (Fig. 3A). Conversely, cv. Cabernet Sauvignon exhibited a less intense pace, with minimal overlap of phases occurring simultaneously. The transition through these stages was gradual and stretched between 3 and 7 DOE, leaving 100% of buds lingering in the swelling phase for more than three days. Eventually, 80% of buds transitioned to BBCH 04–06 phases at 10 DOE (Fig. 3B).

Although the representation in grouped stages limited the ability to examine phenological differences in detail, the last observation carried out in Cabernet Sauvignon (10 DOE) showed no buds at the complete budburst stage; instead, it closely mirrored the condition observed in Chardonnay at a much earlier time (7 DOE). Detailed data are available in Table S2.

### Cold deacclimation monitoring

**Fig. 4:**
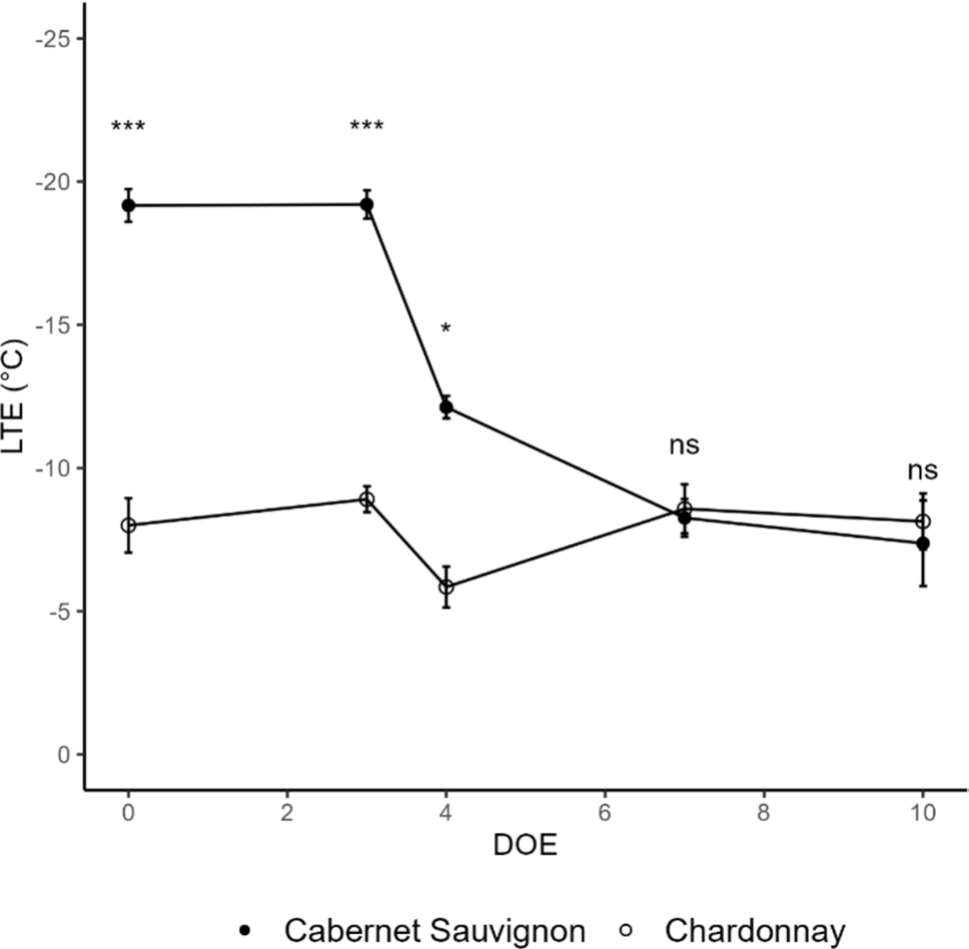
Deacclimation dynamics of cvs. Chardonnay (◦) and Cabernet Sauvignon (•) throughout the experiment. Results are expressed as mean of 3 biological replicates ± standard error. Statistical analyses were performed using one-way ANOVA and Tukey HSD as post hoc test for all pairwise multiple comparison procedures. Statistical significance is represented as *** (*p* < 0.001), ** (*p* < 0.01), * (*p* < 0.05) and ns (not significant). *DOE* day of experiment. *LTEs* Low Temperature Exotherms

Low-temperature exotherms (LTE) were determined in single buds by DTA throughout the experiment as a proxy of deacclimation progression, and statistically different trends were detected in the two varieties (Fig. 4). In Cabernet Sauvignon, LTEs remained stable at around −19 °C during the first two time points and started changing toward less negative values after 3 DOE. Thus, deacclimation for this cultivar appeared to start four days following the transfer to forcing conditions and continued up to 7 DOE, reaching and settling on its lowest levels. In contrast, Chardonnay LTEs did not vary throughout the experiment, suggesting that this cultivar was already deacclimated at 0 DOE. Comparable LTE levels were registered from 7 DOE onwards for both cultivars.

### Differential gene expression

**Fig. 5:**
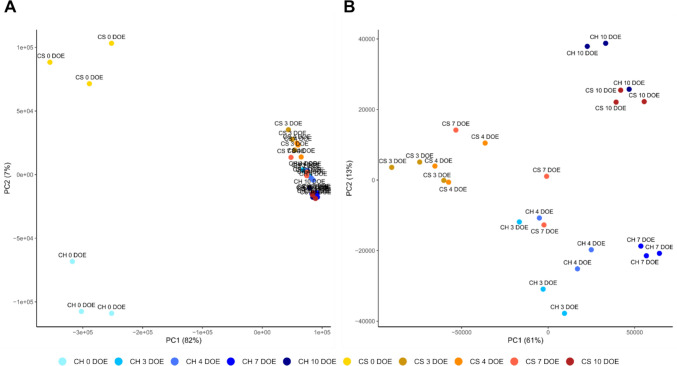
Principal Component Analysis (PCA) of RNA-seq data of Chardonnay (CH) and Cabernet Sauvignon (CS) single-node cuttings throughout the experiment (**A**). To improve sample separation, PCA analysis was also performed excluding 0 DOE samples (**B**). *DOE* day of experiment

To gain insights into the molecular mechanisms underpinning budbreak regulation, high-throughput RNA sequencing was employed to investigate the transcriptome changes in cultivars Chardonnay and Cabernet Sauvignon single-node cuttings at different developmental stages. A Principal Component Analysis (PCA) was performed to assess the quality of replicates and to monitor potential transcriptome changes throughout the experimental time course. PC1 (X-axis) and PC2 (Y-axis) explained 82% and 7% of the variance, respectively. The great variability captured by PC1 explained the segregation of 0 DOE samples of both varieties from all other time points. On the other hand, PC2 drove a clear separation between the 0 DOE samples of Chardonnay and Cabernet Sauvignon, leaving all the other time points less resolved for both varieties (Fig. 5A). To better capture the transcriptomic changes taking place throughout forcing conditions, after 0 DOE, a second PCA was performed excluding this time point (Fig. 5B**)**. The two components were thusly found to explain 61% and 13% of the variance, respectively, and distinct behaviors were observed for the two varieties. Namely, Cabernet Sauvignon samples from 3 to 4 DOE, found to group together, clustered separately from all Chardonnay samples along PC1. The late-budbreak cultivar 7 DOE samples appeared more sparse and closer to early Chardonnay samplings, suggesting a convergence of the transcriptomic landscape of the two cultivars at distinctly different timings. The early-budbreak cultivar 7 DOE samples were segregated separately from all the rest. Despite this different pacing, both varieties reached a similar transcriptome state at the end of the time course (10 DOE), distinguished from the others by PC2. On the other hand, PC1, which separated the samples based on their temporal progression, clearly showed that the samples 7 DOE of Chardonnay and 10 DOE of the two cultivars are nearly corresponding, marking the attainment of a near-stationary state in the early cultivar and its possible initiation in the late one.

Loadings extraction of both PCA analyses was undertaken in an attempt to identify highly discriminating genes among time points. This resulted in the identification of very few genes with a threshold of |loading|> 0.2 (Table S3), indicating that the differences in sample segregation exhibited in both PCAs are the result of a high number of genes which determine the separation among samples in a non-predominant manner relative to one another.

Therefore, despite the low percentage of variance explained by PC2, the different patterns emerging between the two varieties, from both principal components, showed that massive changes in gene expression seemed to be restricted to the very early time points. Eventually, a convergence between the two varieties’ transcriptomes throughout the time course took place.

**Fig. 6:**
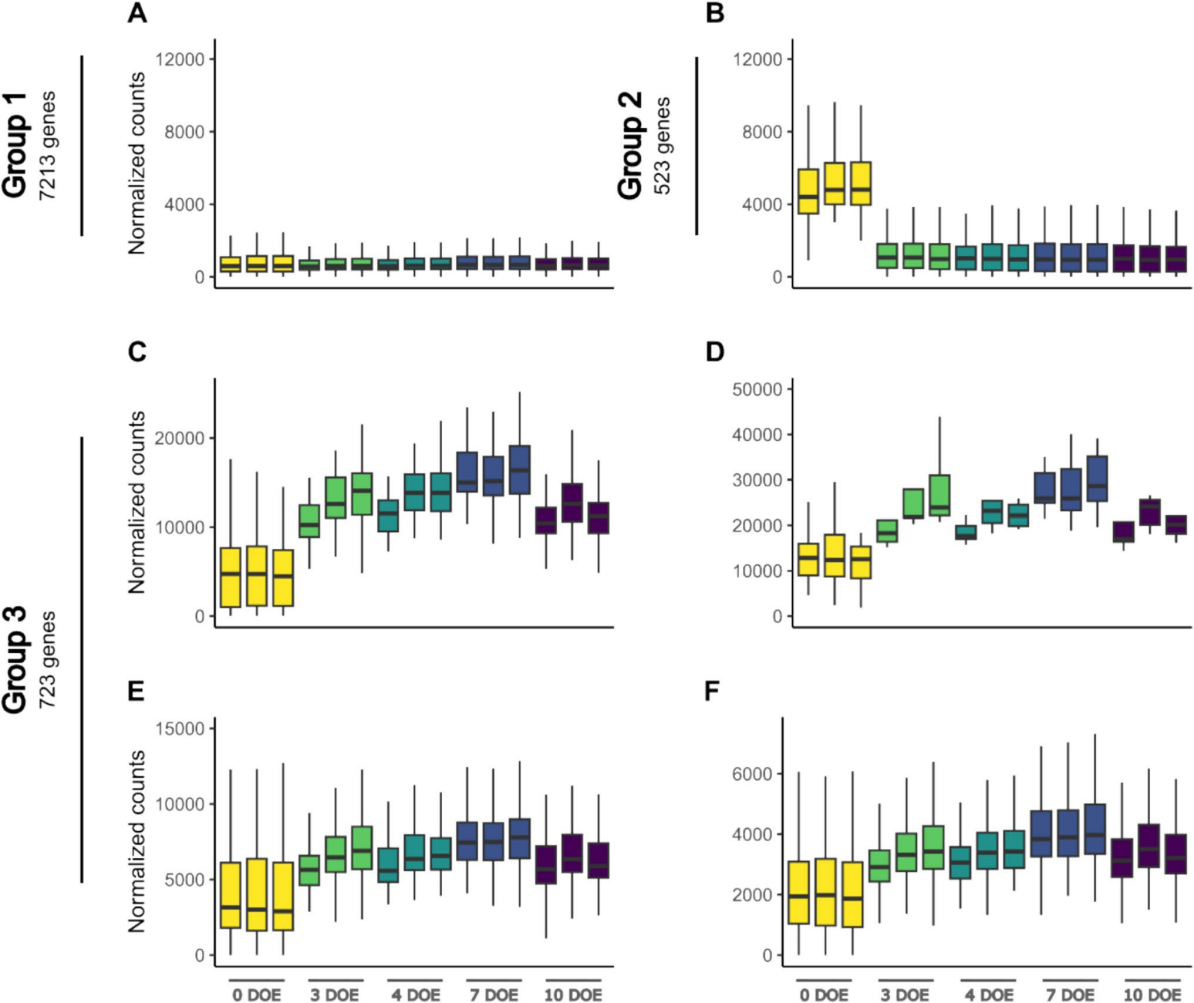
k-means clustering resulting from differentially expressed genes detected in Chardonnay single-node cutting throughout the experiment. Similar clusters were further combined into groups

**Fig. 7:**
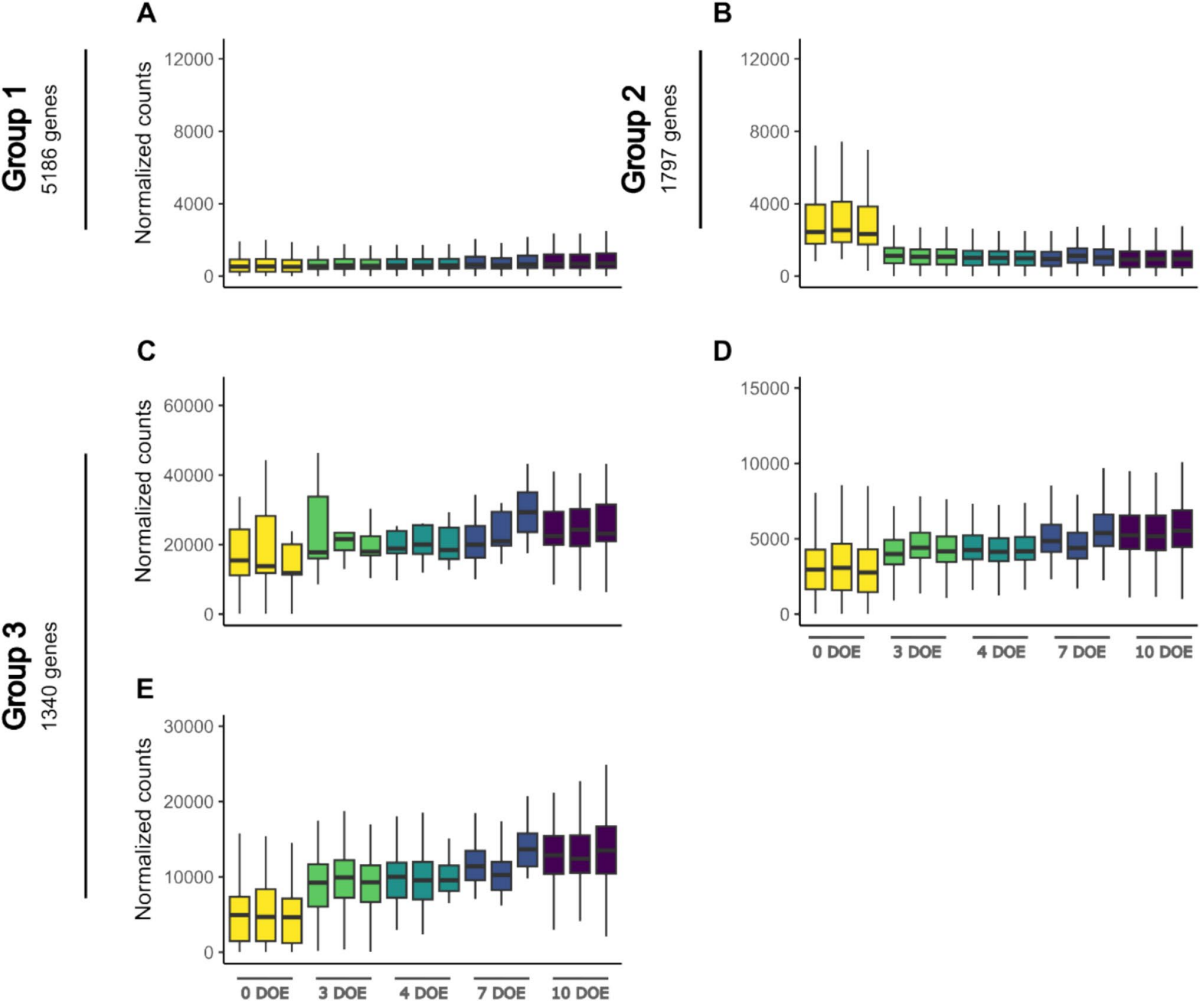
k-means clustering resulting from differentially expressed genes detected in Cabernet Sauvignon single-node cutting throughout the experiment. Similar clusters were further combined into groups

Detected transcripts were considered differentially expressed genes (DEGs) if |log2foldchange|> 1 and padj < 0.05. DEGs count revealed by the comparison between the five-time points are indicated in Table 1. A full list is available in Table S4. The greatest number of DEGs, both upregulated and downregulated, was found in the transition between 0 to 3 DOE in both grapevine cultivars, suggesting an important molecular reprogramming in the transition from 4 °C to forcing conditions. To gather insights into the prevailing gene expression dynamics among sequenced transcripts, a *k*-means clustering was performed based on transcript levels across the time course experiment. The clustering was applied to each cultivar separately to emphasize possible gene groups with distinct expression profiles or magnitude. Ten specific trends were detected in both Chardonnay and Cabernet Sauvignon, grouped according to their pattern. Three major groups were detected for Chardonnay (Fig. 6): group 1, whose gene’s expression does not change substantially throughout the experiment; group 2, including genes whose expression lowers in the transition from 0 to 3 DOE; group 3, containing genes which are increasingly upregulated from 0 to 7 DOE, and slightly downregulated at 10 DOE. Three clearly distinguishable groupings could also be identified for late-budbreak Cabernet Sauvignon (Fig. 7), namely groups 1 and 2, not dissimilar to those described in Chardonnay, and group 3 clustering genes with gradually increasing expression from 0 to 10 DOE (Table S5). A prominent difference between cultivars could be ascribed to a non-symmetrical sorting of genes among the three gene groups. Group 1 comprises the largest set of genes in both cultivars; however, group 1 of stably expressed genes is largest in Chardonnay, while in the late-budbreak Cabernet Sauvignon there are more genes exhibiting the dynamic expression profiles of group 2 and group 3 than in the early-budbreak counterpart (Table S6).

**Table 1:** Differentially Expressed Genes (DEGs) count (|log2foldchange|> 1; padj < 0.05) between time point comparisons

Comparison	Chardonnay		Cabernet Sauvignon	
	UP	DOWN	UP	DOWN
0 DOE vs 3 DOE	11,471	8194	11,190	8067
3 DOE vs 4 DOE	2517	3052	2097	3362
4 DOE vs 7 DOE	4546	3237	3816	4318
7 DOE vs 10 DOE	1866	5239	3552	3346

### Comparative analysis of gene expression

**Fig. 8:**
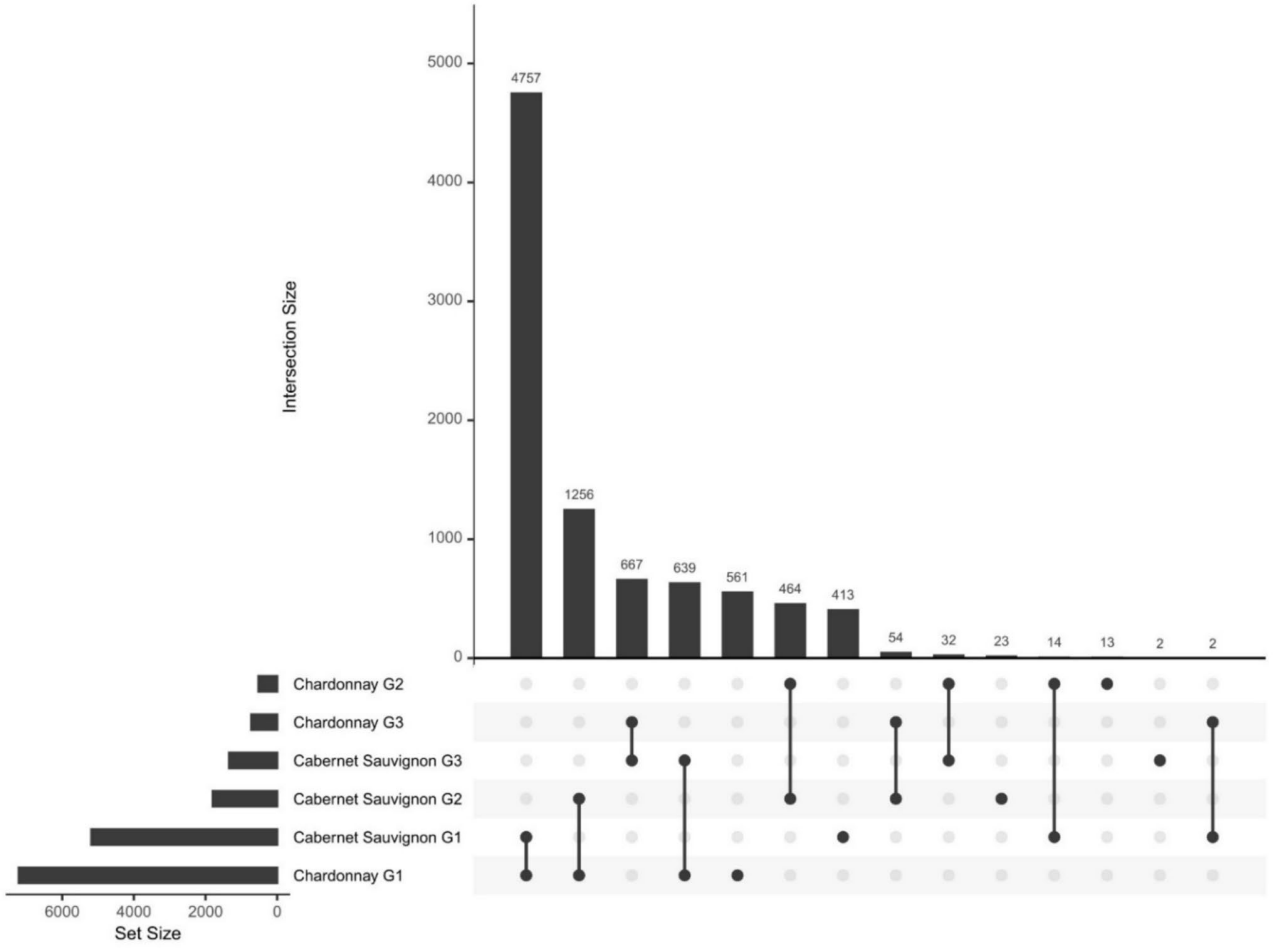
UpSet plot showing gene intersections in Chardonnay and Cabernet Sauvignon cluster groups. G1 = group 1, G2 = group 2; G3 = group 3

To visualize the similarities and discrepancies in gene expression dynamics, the previously described gene groups were intersected, and an UpSet plot was generated (Fig. 8). The most conspicuous number of shared genes was detected in the comparison between Chardonnay group 1 and Cabernet Sauvignon group 1, with 4747 genes stably expressed across time points with minor variation.

The second most represented intersection occurs between Chardonnay group 1 and Cabernet Sauvignon group 2, indicating 1256 genes which lower their expression in the late-budbreak cultivars from 0 to 2 DOE while remaining stable in the early-budbreak one. The intersection between Chardonnay and Cabernet Sauvignon groups 3 followed with 667 common genes that increase their expression up to 7 DOE in the first and up to 10 DOE in the latter. The fourth largest intersection involved Chardonnay group 1 and Cabernet Sauvignon group 3 with 639 genes. Lastly, 464 shared accessions were detected in the comparison between group 2 of the two varieties. Smaller intersections encompassing few genes are present and may be considered spurious.

To better assess the relative value of the observed intersections, the relative importance of shared genes while accounting for the size of the groups being compared, was calculated (ratio of shared vs merged genes) (**Table S7**). The highest ratio (62%) was found for the intersection between groups 1 of both cultivars, followed by the intersection between groups 3 (48%), further highlighting the relevance of this overlapping. In contrast, the intersection involving group 2 of both cultivars showed a lower ratio (25%), indicating a smaller degree of overlap relative to the group sizes. Interestingly, while the intersection between Cabernet Sauvignon group 2 and Chardonnay group 1 was numerically the second largest, its normalized ratio was relatively low (16%). Similarly, the intersection between Cabernet Sauvignon group 3 and Chardonnay group 1 had a modest ratio (8%), reflecting limited overlap despite the group sizes.

**Fig. 9:**
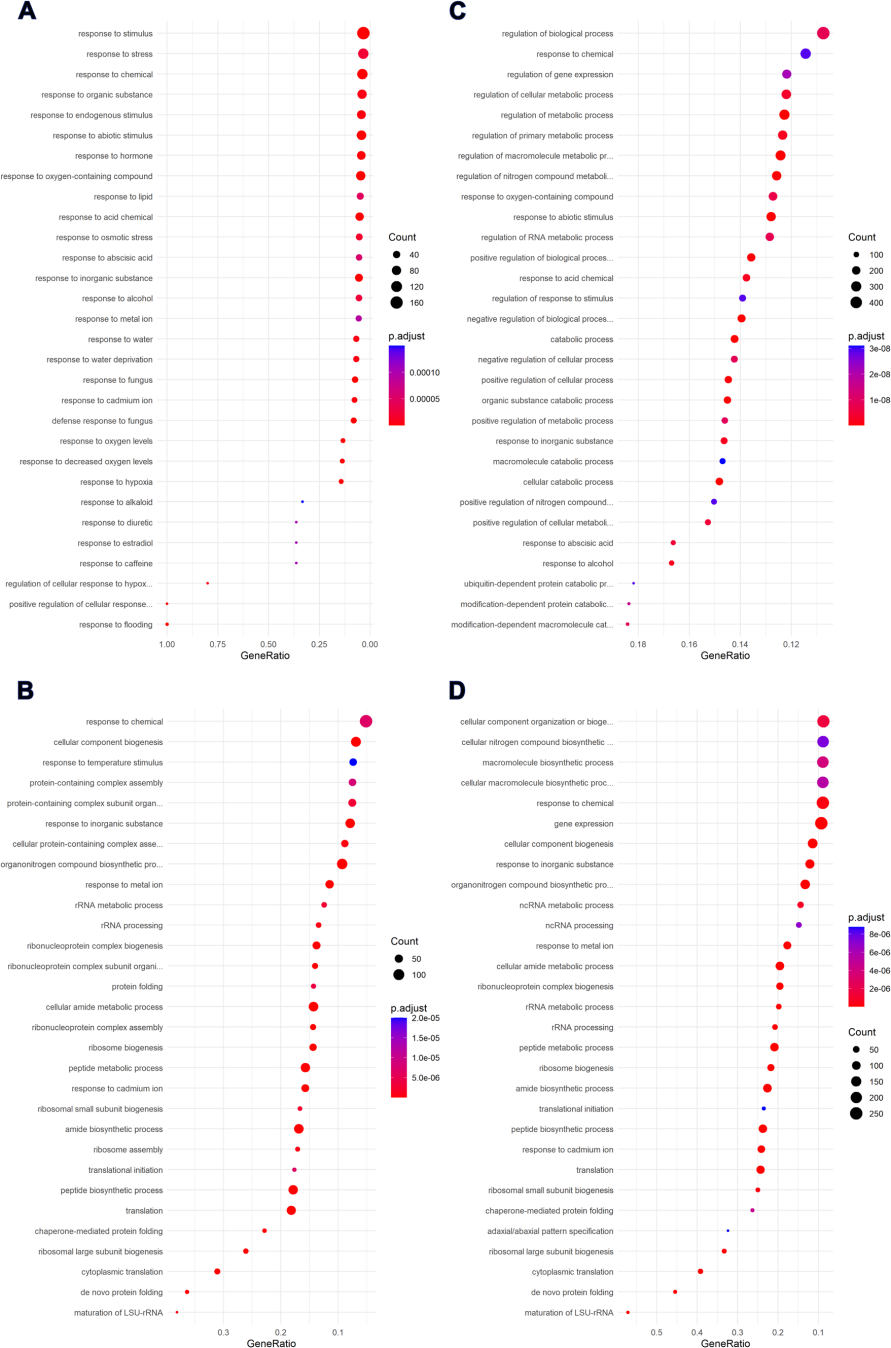
Gene Ontology enrichment analysis of biological processes taking place in Chardonnay and Cabernet Sauvignon single-node cuttings throughout the experiment. The top 30 most significant BP terms (*p* < 0.001) are shown for each gene group. **A** Chardonnay group 2, **B** Chardonnay group 3, **C** Cabernet Sauvignon group 2, **D** Cabernet Sauvignon group 3

To further characterize the differences between Chardonnay and Cabernet Sauvignon gene clusters, Gene Ontology enrichment analysis was performed with a focus on the ‘Biological Processes’ (BP). Significantly enriched BP terms were determined (*p*-value < 0.05) and the top 30 most significant terms for gene groups 2 and 3 are summarized in Fig. 9.

BP terms in groups 2, representing genes whose expression is higher at 0 DOE compared to the rest, differed between the two varieties. Most terms found in Chardonnay were related to water (e.g. ‘response to flooding’, ‘response to water’), hypoxia (e.g. ‘regulation of cellular response to hypoxia’, ‘response to decreased oxygen levels’) and hormonal stimuli (e.g. ‘response to abscisic acid’, ‘response to hormone’), among other terms more generally related to stress responses (Fig. 9A). With the exception of the highly significant ‘response to abscisic acid’ term, no clear branch of metabolism was identifiable among the BP terms found in Cabernet Sauvignon, apart from indications of ongoing protein catabolic processes (e.g. ‘ubiquitin-dependent protein catabolic process’, ‘macromolecule catabolic process’) (Fig. 9C).

When it comes to groups 3, clustering genes whose expression increased from 0 to 7 DOE in Chardonnay (Fig. 9B) and up to 10 DOE in Cabernet Sauvignon (Fig. 9D), striking similarities could be observed. Most BP terms found in each variety were related to protein synthesis and folding, as well as ribosome biogenesis and assembly (e.g. ‘maturation of LSU-rRNA’, ‘de novo protein folding’, ‘translation’). Few unique terms were apparent, such as ‘response to temperature stimulus’ which stood out in Chardonnay, whereas terms concerning gene expression, non-coding RNAs (e.g. ‘ncRNA processing’), and tissue regionalization (e.g. adaxial/abaxial pattern specification’) were noted in Cabernet Sauvignon.

All BP terms significantly enriched in group 1 of both cultivars indicated an increase in cell metabolism concerning gene expression, protein synthesis and organelle organization **(**Figure S2**).**

### Expression analysis of dormancy-related genes

Dormancy-markers genes *VviNCED6* (Vitvi05_01chr02g19450) (previously named *VviNCED1* by Zheng et al. (2015)), *VviSVP2* (Vitvi05_01chr18g07640) and *VviDRM1* (Vitvi05_01chr10g08760) as well as *VviFT* (Vitvi05_01chr08g17740), associated to dormancy interruption, were searched for among DEG data and their normalized gene counts used to determine their expression (Figure S3). The BLAST search successfully identified a homolog of *DRM1* in the *V. vinifera* reference genome; specifically, Vitvi05_01chr10g08760 emerged as a most alike sequence.

**Fig. 10:**
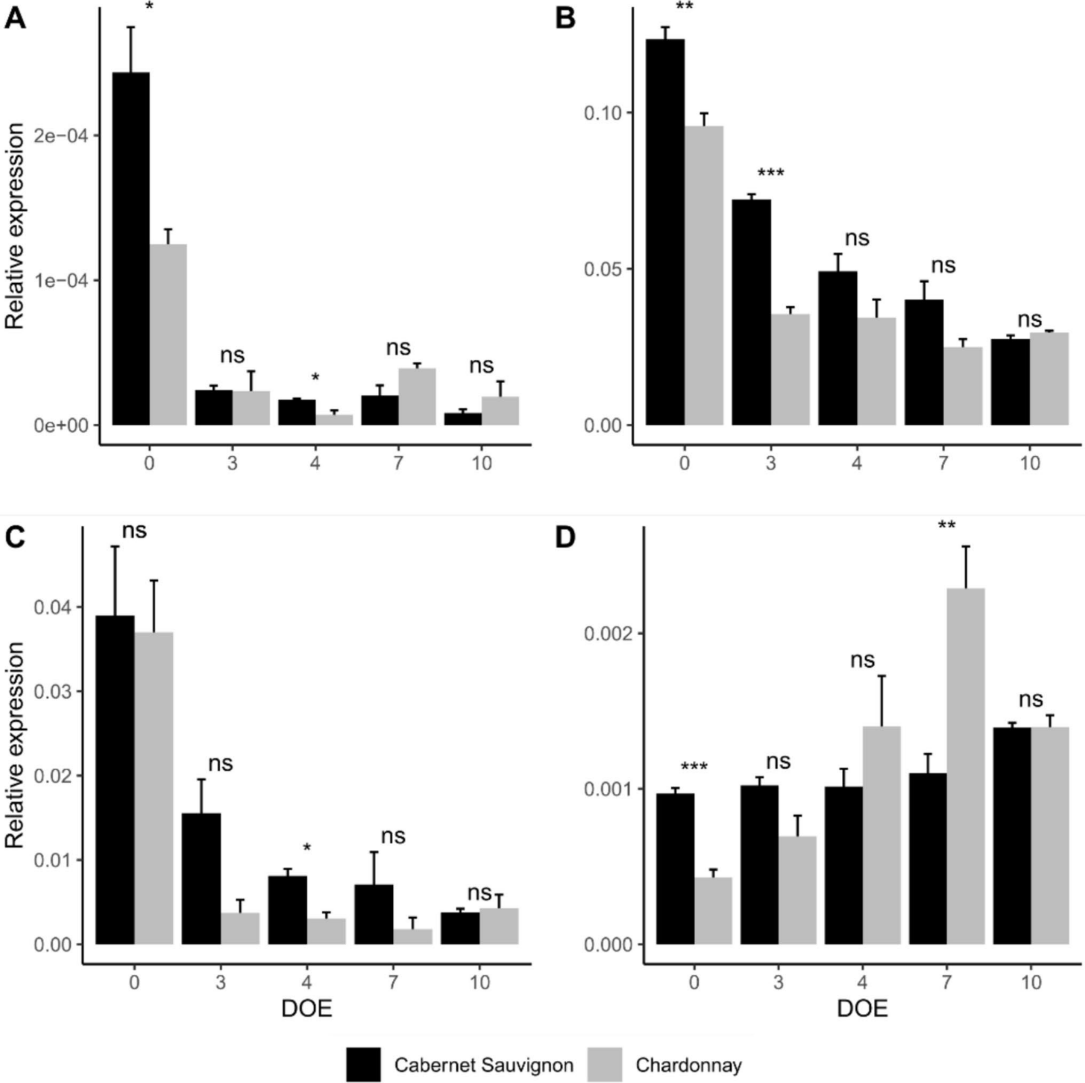
Relative expression of genes *VviNCED6* (**A**), *VviSVP2* (**B**), *VviDRM1* (**C**), *VviFT* (**D**) in cv. Cabernet Sauvignon (black bars) and cv. Chardonnay (grey bars) throughout the experiment. DOE = day of experiment. Results are expressed as a mean of 3 biological replicates ± standard error. Statistical analyses were performed using one-way ANOVA and Tukey HSD as post hoc test for all pairwise multiple comparison procedures. Statistical significance is represented as *** (*p* < 0.001), ** (*p* < 0.01), * (*p* < 0.05) and ns (not significant). *VviNCED6* = *Nine-cis-Epoxycarotenoid Dioxygenase 6*; *VviSVP2* = *Short Vegetative Phase 2*; *VviDRM1* = *Dormancy Associated Protein-Like 1*; *VviFT* = *Flowering Locus T*

For a more precise quantification of gene expression, normalized gene count data were validated by qRT-PCR (Fig. 10). Striking differences were detected at 0 DOE in the expression of dormancy marker gene *VviNCED6*, with higher levels of expression in late-budbreak cultivar Cabernet Sauvignon compared to early-budbreak Chardonnay. A decrease was found in both varieties starting from 3 DOE (Fig. 10A). These results are consistent with the potentially higher ABA levels in the late-budbreak variety, further supported by other members of the ABA biosynthetic pathway including grapevine *VviNCED4* and *VviNCED5* found in the T2T genome (Figure S4).

Decreasing trends were also recorded for dormancy-associated *VviSVP2* in both cultivars, with highly significant differences among varieties from 0 to 3 DOE. In this case, the expression pattern of *VviSVP2* observed in Cabernet Sauvignon was analogous to that reported for *VviNCED6*. In Chardonnay, although the expression levels were significantly different from Cabernet Sauvignon at the initial time points, the expression pattern showed a decreasing trend from 3 DOE before stabilizing (Fig. 10B).

A similar decreasing expression pattern was successfully detected for the putative *VviDRM1* in buds of both Chardonnay and Cabernet Sauvignon. The reduction was apparent from 3 DOE for both genotypes with a sharper decline in Chardonnay, in which the expression remained stably low henceforth, and a smoother declining trend in Cabernet Sauvignon (Fig. 10C).

Lastly, the gene *VviFT* presented significant differences in its expression comparing the two varieties. Specifically, *VviFT* expression remained constant from 0 to 10 DOE in late-budbreak Cabernet Sauvignon, while in the early-budbreak Chardonnay the transcription started from lower values at 0 DOE and gradually increased to reach a peak at 7 DOE, following which its expression decreased (Fig. 10D).

## Discussion

The molecular machinery of dormancy regulation remains largely unknown, and the effort required to monitor in the field this natural process, spanning several months and subjected to seasonal variability, hinders the collection of reliable data. For these reasons, the possibility to model dormancy progression and reduce the influence of environmental factors is desirable. The use of cuttings has proven to be a convenient and efficient way to carry out experiments on a smaller scale before applying findings to entire vineyards. It allows the comparison of different grape varieties or clones under standardized conditions, helping in identifying genetic factors that contribute to budbreak precocity and dormancy release (Fila et al. 2012; Camargo Alvarez et al. 2018). Our results showed that the single-node cuttings model has been effective under forcing conditions and, notably, in an experiment where the temporal shift between two cultivars was crucial. In our experiment, the expected budbreak timing for the two genotypes was confirmed in cuttings subjected to standardized conditions: more than 50% of Chardonnay buds reached the BBCH 07–09 stages at 10 DOE, with Cabernet Sauvignon achieving the same stages four days thereafter (data not shown). This is in alignment with natural budbreak timings observed in the field (Camargo Alvarez et al. 2020), while keeping in consideration that cuttings treated with stable chilling temperatures tend to reach budburst more rapidly than field-collected material (North and Kovaleski 2023). Visual phenotyping is supported by DTA data, which clearly depict the different evolutions of cold hardy states in the two genotypes in response to forcing conditions and confirm the slower reactivity of late-budbreak Cabernet Sauvignon, also observed in field conditions. Interestingly, Chardonnay LTEs, which correspond to freezing events of intracellular water within buds, routinely used as indicators of cold hardiness levels, remained unchanged throughout the time of observation. This may be due to a different sensitivity and perception of chilling temperatures between the two varieties. On the other hand, temperatures that are adequately low to maintain buds in a cold-hardy state in one cultivar, may contribute to chilling accumulation and induce deacclimation in another (North and Kovaleski 2023).

The *k*-means clustering results further support the enhanced metabolic activity of Chardonnay cuttings, anticipated as compared to Cabernet Sauvignon. In fact, two of the clusters, identified as group 3 (G3) in both cultivars, included genes with an increasing expression trend over time which peaked earlier in Chardonnay at 7 DOE, compared to Cabernet Sauvignon, where it peaked at 10 DOE. This temporal shift could suggest an earlier response in Chardonnay compared to Cabernet Sauvignon, potentially reflecting cultivar-specific differences in transcriptional reprogramming and physiological responses. In fact, out of a total number of 1340 in Cabernet Sauvignon and 723 in Chardonnay (Table S2), 667 genes were found to be shared between the two sets (Fig. 8). Functional annotation further supports the observation of a consistent overlap in biological processes within these gene sets, predominantly involving protein synthesis and folding. These processes, critical for cellular function, have been shown to play key roles in various developmental stages, including budbreak and growth resumption (Zhang et al. 2016).

Moreover, a distinct distribution of genes across the three groups in the two cultivars was observed. In detail, several genes assigned to groups 2 and 3 in Cabernet Sauvignon, which exhibit variable expression patterns over the time course, belong to group 1 and maintain a steady expression level across all time points in Chardonnay. Thus, these genes could be actively modulated in the late budbreak cultivar in view of budbreak but no longer play a primary role in the early budbreak one.

In addition to large-scale analysis, and to confirm its reliability, a targeted and more detailed study was conducted on genes identified in the literature as associated with dormancy progression. The expression pattern of the ABA-synthesis enzyme *VviNCED6* appeared consistent with the responses of the two cultivars to forcing conditions. Indeed, the gene was more highly expressed at the first sampling in Cabernet Sauvignon, as shown by qPCR data and strengthened by normalized RNA-seq counts. On the other hand, the role in the natural dormancy release of *VviNCED6* was previously supported by Zheng et al. (2015), who referred to it as *VviNCED1*. Since ABA production is strongly associated with a dormant state in buds (Zheng et al. 2015), this evidence supports an anticipated dormancy release in Chardonnay. This result is further confirmed by the expression trends of two other *NCEDs*, *VviNCED4* and *VviNCED5*, and concurs with previously collected evidence showing a decrease of *VviNCED4* expression in buds collected from January to March in field conditions in the northern hemisphere (Shangguan et al. 2020). Furthermore, *VviNCED4* gene counts consistently maintain a low level in early-budbreak Chardonnay throughout the experiment, suggesting that its peak expression may have occurred prior to the initial sampling. Dormancy-regulated MADS-box gene *VviSVP2* is also most expressed at 0 DOE in both cultivars; however, its decreasing trend appears sharper in Chardonnay and more gradual in Cabernet Sauvignon. Previous evidence has described a reduction of *VviSVP2* expression in the transition from paradormancy to endodormancy in grapevine buds, but no clear trend was detected during ecodormancy release in forcing conditions (Vergara et al. 2021). Nevertheless, kiwifruit *SVP2* overexpression was found to delay budbreak in *A. deliciosa* and, in association with a proposed ABA-mimicking effect from a transcriptomic point of view, lead to connecting *AdSVP2* to deep bud dormancy maintenance (Wu et al. 2017). In light of these considerations, the *VviSVP2* patterns observed in this study support an anticipated release from dormancy in Chardonnay, in accordance with what observed for *VviNCED6* and *VviNCED4*. Moreover, it is rather reasonable that the cessation of ABA hormone synthesis precedes the induction of budbreak, in which VviSVP could play a pivotal role.

The contrasting relation between *SVP* and the flowering time regulator *FT* has been mostly characterized in *A. thaliana* (Jeong et al. 2007). However, recently collected data have suggested a similar relationship between *VviFT* and *VvDAM3-SVP* in grapevine, across several experimental conditions (Vergara et al. 2021). Moreover, *VviFT* was found to be downregulated in grapevine buds and leaves exposed to short photoperiod (Vergara et al. 2016), and in buds during endodormancy (Díaz-Riquelme et al. 2012; Vergara et al. 2016). Additionally, H_2_CN_2_ prompted *VviFT* expression in grapevine buds (Vergara et al. 2016) similarly to natural budbreak and hypoxia (Vergara et al. 2012). In this study, differential *VviFT* expression patterns were observed in late-budbreak cultivar Cabernet Sauvignon and early-budbreak Chardonnay. *VviFT* expression in Chardonnay sharply increases during forcing conditions to reach a peak at 7 DOE, corresponding to more than 50% of buds having reached the stages of initial green growth, following which its expression decreases. In these phases of phenological development, grapevine buds show clearly visible morphological changes and are completely deacclimated. Although less noticeable, a slight increase of expression is also present in Cabernet Sauvignon at 10 DOE, allowing us to speculate that Cabernet Sauvignon might show a similar peak at a later stage. These findings hint at the involvement of *VviFT* in the preparatory stages of budbreak, kickstarting the overall process in concomitance with the perception of growth-promoting temperatures and culminating during wool emergence. The observed decrease of expression at more advanced BBCH stages suggests that the control of subsequent bud and shoot development may be handed over to other, currently unknown, molecular drivers. The less clear trend observed in Cabernet Sauvignon could be explained by the slower reactivity demonstrated by LTEs, as well as previously described gene expression evidence.

Finally, to our knowledge, this study presents the first example of a putative homolog of *DRM1* identified in grapevine. *AdDRM1* was found to exhibit a strong inverse correlation with growth onset in kiwifruit and to be downregulated by dormancy-breaking compound H_2_CN_2_ (Wood et al. 2013). In addition, a connection to ABA and auxin signaling was hypothesized by Stafstrom (2000), following the detection of a relevant reduction of *PsDRM1/2* expression in pea axillary buds after terminal bud removal, and induction by ABA treatment. Similarly, the expression of *DRM/ARP* gene *RpARP1* was downregulated by exogenous auxin application in *Robinia pseudoacacia* and was negatively correlated to hypocotyl growth (Park and Han 2003). At present, however, the connection between these hormones and *DRMs* regulation appears controversial and up for debate (Rae et al. 2013). All evidence considered, the expression of putative *VvDRM1* is consistent with its expected role as a dormancy marker. Similarly to what happens for *VviSVP*, it could be speculated that *VviDRM1* also shows high initial levels followed by downregulation, with a swifter decline in Chardonnay and a more gradual decrease in Cabernet. In this perspective, *VviDRM1*, like *VviSVP*, would also act downstream ABA signal, in allowing dormancy release and the resumption of growth.

## Conclusions

The use of single-node cuttings proved to be an effective method for the screening of budbreak timing in grapevine. This method provides a controlled environment for comparison among different grape genotypes and the possibility to quickly phenotype the spring phenology under controlled conditions. This approach allowed us, on the one hand, to exclude the effect of environmental variables and, on the other hand, to distinguish between general regulatory mechanisms that control budbreak and those specific to particular cultivars. One of the key findings of this study is the identification of specific Gene Ontology (GO) categories displaying similar, yet temporally shifted expression patterns between the two cultivars. This suggests that, while the same biological processes may be involved in budbreak across cultivars, their regulation differs, potentially reflecting cultivar-specific timing in dormancy release. Among the genes identified in the literature as crucial for dormancy progression, *VviFT* appears to be the only one exhibiting an expression pattern consistent with the aforementioned shift. In detail, our results confirm the primary role of ABA biosynthesis inhibition for the resumption of growth and budbreak. Other molecular drivers, such as *VviSVP2*, *VviFT* and *VviDRM1*, which, as far as we know, have been little or never studied in grapevine buds, could act downstream. In particular, while *VviSVP2* and *VviDRM1* could be considered “repressors”, as their downregulation is needed for phenological progression, *VviFT* could have a triggering role in the process leading to budbreak.

**Fig. 11:**
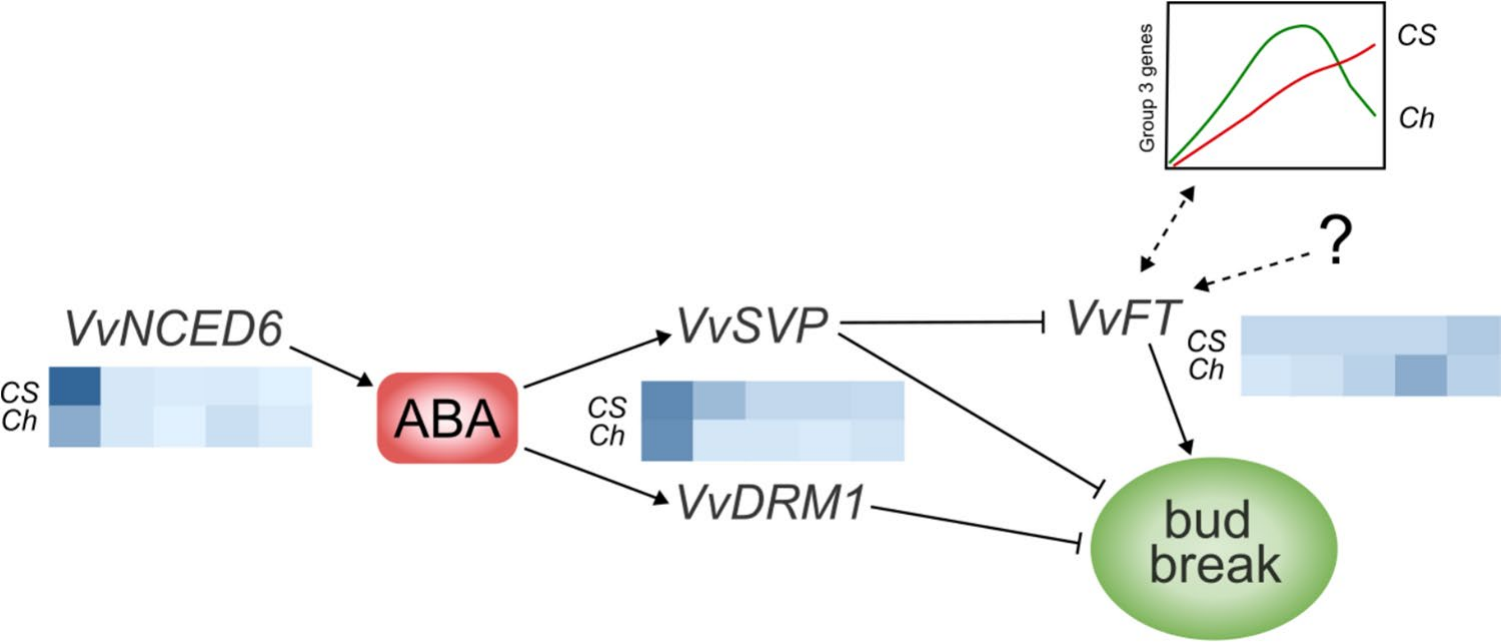
Hypothesis on gene regulatory mechanisms underlying differential budbreak timing. The decrease in ABA and the downregulation of *VviNCED6* are necessary but not sufficient conditions to initiate budburst; in Chardonnay this process appears to be at a significantly more advanced stage. The genes *VviSVP* and *VviDRM1*, possibly controlled by ABA, also show different expression dynamics in the two cultivars, with Chardonnay displaying a more sudden downregulation. This, along with other factors, likely contributes to the activation of *VviFT*, which in Chardonnay peaks in synchrony with budbreak. The concurrent activation of several gene categories (Group 3 in the schematic graph) may be involved in the process. Blue color gradients are purely illustrative and represent the gene expression patterns, measured by qRT-PCR throughout the experiment (from 0 to 10 DOE). For simplicity, the same representation has been used for *VviSVP* and *VviDRM1*. *Ch* Chardonnay, *CS* Cabernet Sauvignon

Moreover, our findings revealed that the activation of *VviFT* is not solely dependent on the downregulation of *VviSVP* (similar in both Chardonnay and Cabernet Sauvignon), indicating that other cultivar-specific factors play a role in *VviFT* activation. The focus of this study does not extend to establishing a direct cause-and-effect relationship between the expression of genes such as *VviFT* and the broader metabolic shifts described by Group 3 (G3); the complexity of these interactions requires more detailed investigation to distinguish between regulatory effects and downstream responses (Fig. 11). However, our results enhance the understanding of the intricate regulatory network controlling dormancy in grapevine buds and provide a reliable method for further investigations in this field.

## Supplementary Information

Below is the link to the electronic supplementary material.

Supplementary file1 (DOCX 560 KB)

Supplementary file2 (XLSX 40061 KB)

Supplementary file3 (XLSX 189 KB)

## Data Availability

RNA-Seq data were deposited into the Sequence Read Archive (SRA) database under BioProject ID PRJNA1240876.
