# Supplementary material for: Modeling budbreak precocity in grapevine: insights from comparative gene expression analysis in single-node cuttings
Source: Planta. 2025 Apr 5;261(5):104. doi: 10.1007/s00425-025-04677-2 (PMC11972195; doi:10.1007/s00425-025-04677-2)
Supplement: Supplementary file 1 — Supplementary file1 (DOCX 560 KB) [file 425_2025_4677_MOESM1_ESM.docx]

**Supplementary materials**

**Table S1**. List of primers used for qPCR analysis.

| **Target** | **T2T accession** | **Primer sequence (5’-3’)** | | **Amplicon length** |
| --- | --- | --- | --- | --- |
| *VvNCED6* | Vitvi05_01chr02g19450 | F | CTGGGAAGGAGAACACGGTA | 101 bp |
|  |  | R | TTGGTTACCTCTCCGGTCAC |  |
| *VvSVP2* | Vitvi05_01chr18g07640 | F | CGGCTAATGACTATGGAATCGG | 130 bp |
|  |  | R | GCTTGAGAGATGTGTCAGAGC |  |
| *VvDRM1* | Vitvi05_01chr10g08760 | F | CTATGACTCCCACCACTCCC | 174 bp |
|  |  | R | GGAGATGAACAGGCGTAGGT |  |
| *VvFT* | Vitvi05_01chr08g17740 | F | AGAAACGCAGACAGACGGTT | 128 bp |
|  |  | R | AGCTGTTTCTCGCTGAGCAT |  |

**Table S2**. Detailed phenotypic data on Chardonnay (CH) and Cabernet Sauvignon (CS) single-node cuttings in forcing conditions classified as per BBCH scale. DOE = day of experiment.

|  | **0 DOE** | | **3 DOE** | | **4 DOE** | | **7 DOE** | | **10 DOE** | |
| --- | --- | --- | --- | --- | --- | --- | --- | --- | --- | --- |
|  | **CH** | **CS** | **CH** | **CS** | **CH** | **CS** | **CH** | **CS** | **CH** | **CS** |
| **BBCH 00** | 100% | 100% | 0% | 40% | 0% | 0% | 0% | 0% | 0% | 0% |
| **BBCH 01 – 03** | 0% | 0% | 80% | 60% | 80% | 100% | 20% | 100% | 20% | 20% |
| **BBCH 04 – 06** | 0% | 0% | 20% | 0% | 20% | 0% | 80% | 0% | 20% | 80% |
| **BBCH 07 – 09** | 0% | 0% | 0% | 0% | 0% | 0% | 0% | 0% | 40% | 0% |
| **BBCH 10** | 0% | 0% | 0% | 0% | 0% | 0% | 0% | 0% | 20% | 0% |

**Table S3**. Loadings extracted from PCA analyses. Loading threshold was set at |loading| > 0.2.

|  | **T2T accession** | **Annotation** |
| --- | --- | --- |
| **0 DOE included** | Vitvi05_01chr04g19030 | *VviDHN1* (Dehydrin 1) |
|  | Vitvi05_01chr07g05590 | *VviSUSY3* (Sucrose Synthase 3) |
|  | Vitvi05_01chr08g06180 | Pyruvate decarboxylase |
|  | Vitvi05_01chr01g20950 | *VviGAPC1* (Glyceraldehyde-3-Phosphate Dehydrogenase) |
|  | Vitvi05_01chr04g24030 | *VviADH1* (Alcohol Dehydrogenase) |
|  | Vitvi05_01chr19g01900 | D-Mannose Binding Lectin Protein |
|  | Vitvi05_01chr19g01920 | Mannose Binding Lectin1 |
|  | Vitvi05_01chr19g09420 | Glycine rich protein family |
| **0 DOE excluded** | Vitvi05_01chr05g10840 | Unknown |
|  | Vitvi05_01chr08g13760 | Metallothionein |
|  | Vitvi05_01chr11g00510 | *VviSUSY4* (Sucrose Synthase 4) |
|  | Vitvi05_01chr19g09420 | Glycine rich protein family |

**Table S6**. Total number of genes included in each cluster group.

| **Cluster** | **Chardonnay** | **Cabernet Sauvignon** |
| --- | --- | --- |
| Group 1 | 7213 | 5186 |
| Group 2 | 523 | 1797 |
| Group 3 | 723 | 1340 |

**Table S7**. Total number of genes included in each cluster group. CS = Cabernet Sauvignon; CH = Chardonnay. G1 = group 1; G2 = group 2; G3 = group 3.

| **Comparison** | **Intersection** | **Union** | **(Intersection/Union)*100** |
| --- | --- | --- | --- |
| CS G1 v CH G1 | 4757 | 7642 | 62% |
| CS G2 v CH G1 | 1256 | 7754 | 16% |
| CS G3 v CH G3 | 667 | 1396 | 48% |
| CS G3 v CH G1 | 639 | 7914 | 8% |
| CS G2 v CH G2 | 464 | 1856 | 25% |


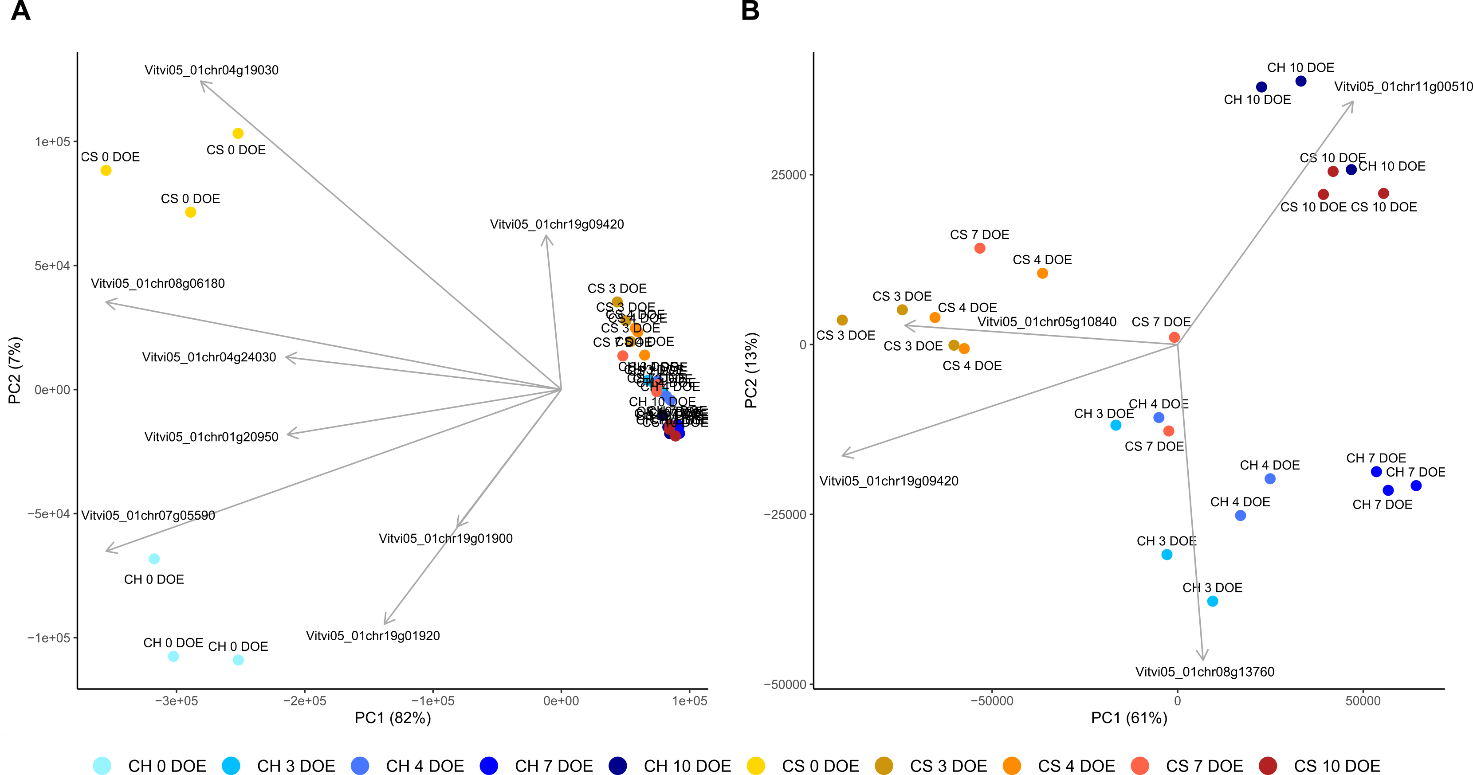


**Figure S1**. Principal Component Analysis of RNA-seq data of Chardonnay (CH) and Cabernet Sauvignon (CS) single-node cuttings throughout the experiment (**A**). To improve sample segregation, PCA analysis was also performed excluding 0 DOE samples (**B**). Loadings were extracted with a | loading | > 0.2 threshold. DOE = day of experiment.


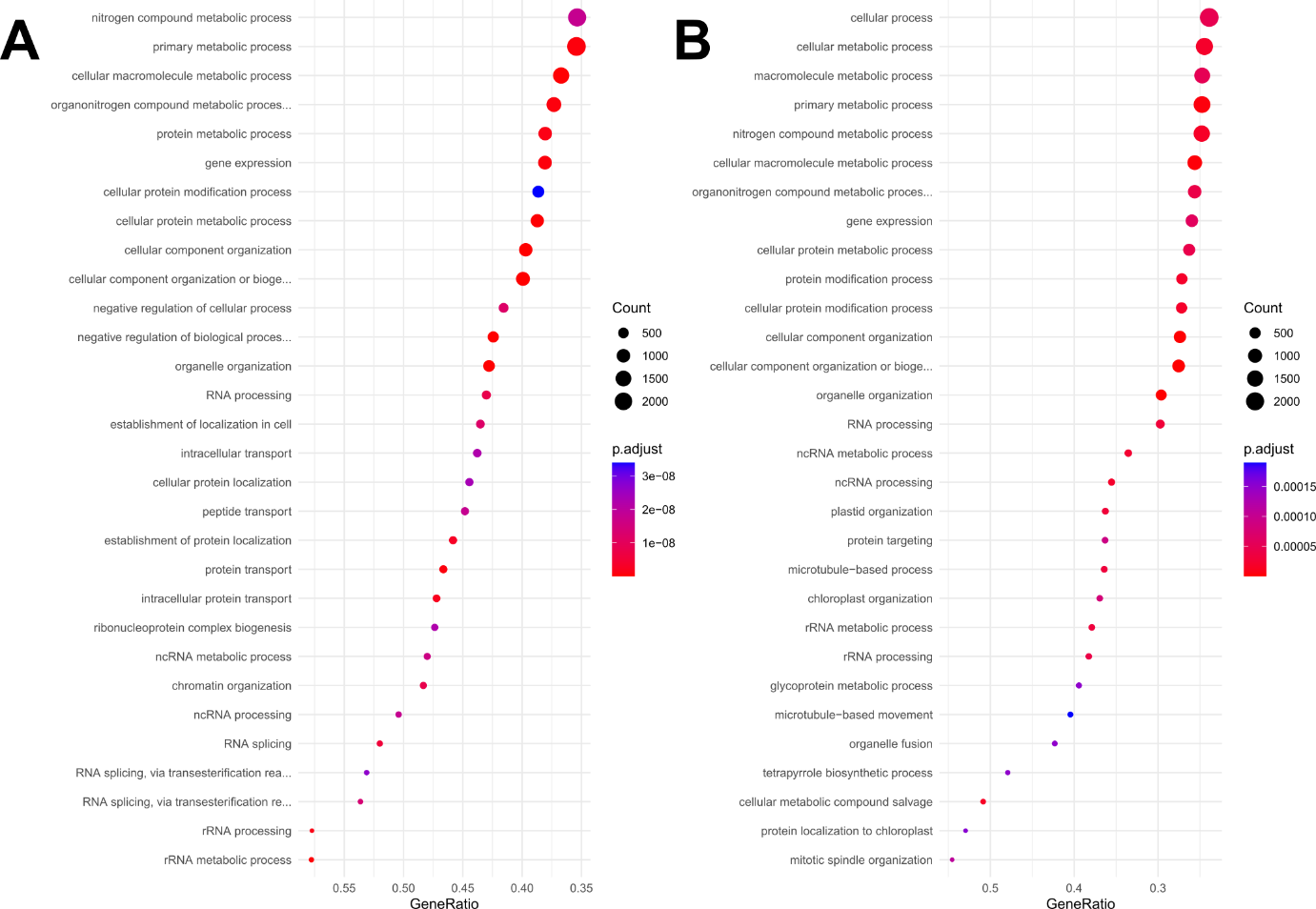


**Figure S2**. Gene Ontology enrichment analysis of biological processes taking place in Chardonnay and Cabernet Sauvignon single-node cuttings throughout the experiment. **A** = Chardonnay group 1, **B** = Cabernet Sauvignon group 1.


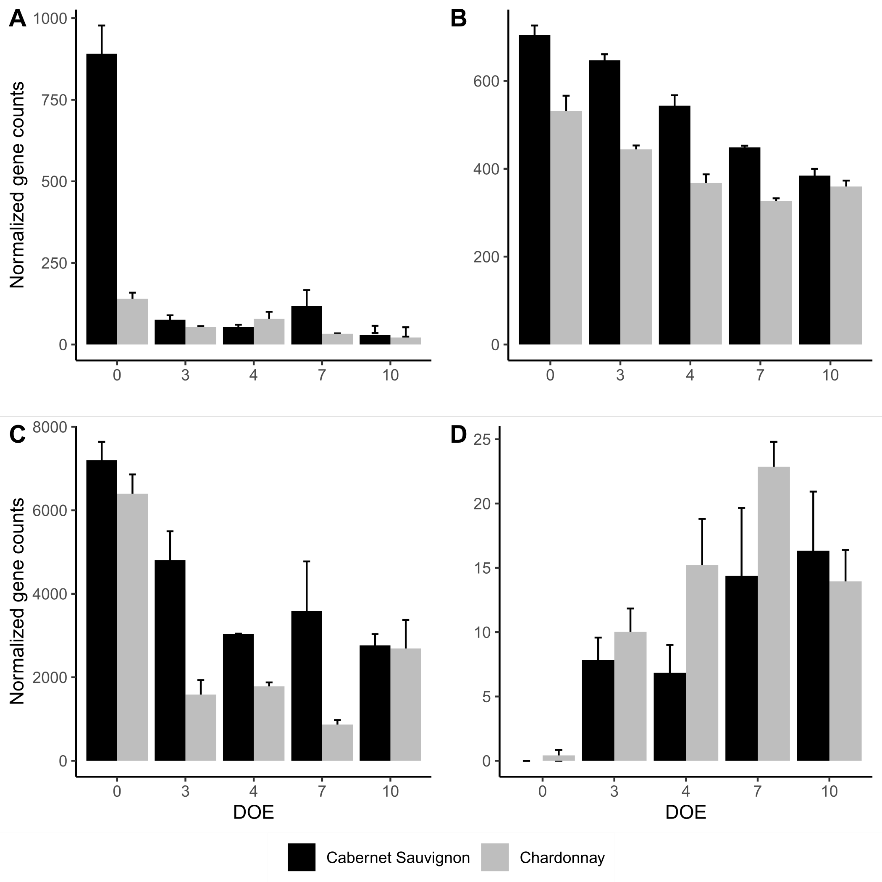


**Figure S3**. DESeq2 normalized gene counts of genes *VvNCED6* (**A**), *VviSVP2* (**B**), *VviDRM1* (**C**), *VviFT* (**D**) in cv. Cabernet Sauvignon (black bars) and cv. Chardonnay (grey bars) throughout the experiment. DOE = day of experiment. Results are expressed as mean of 3 biological replicates ± standard error.


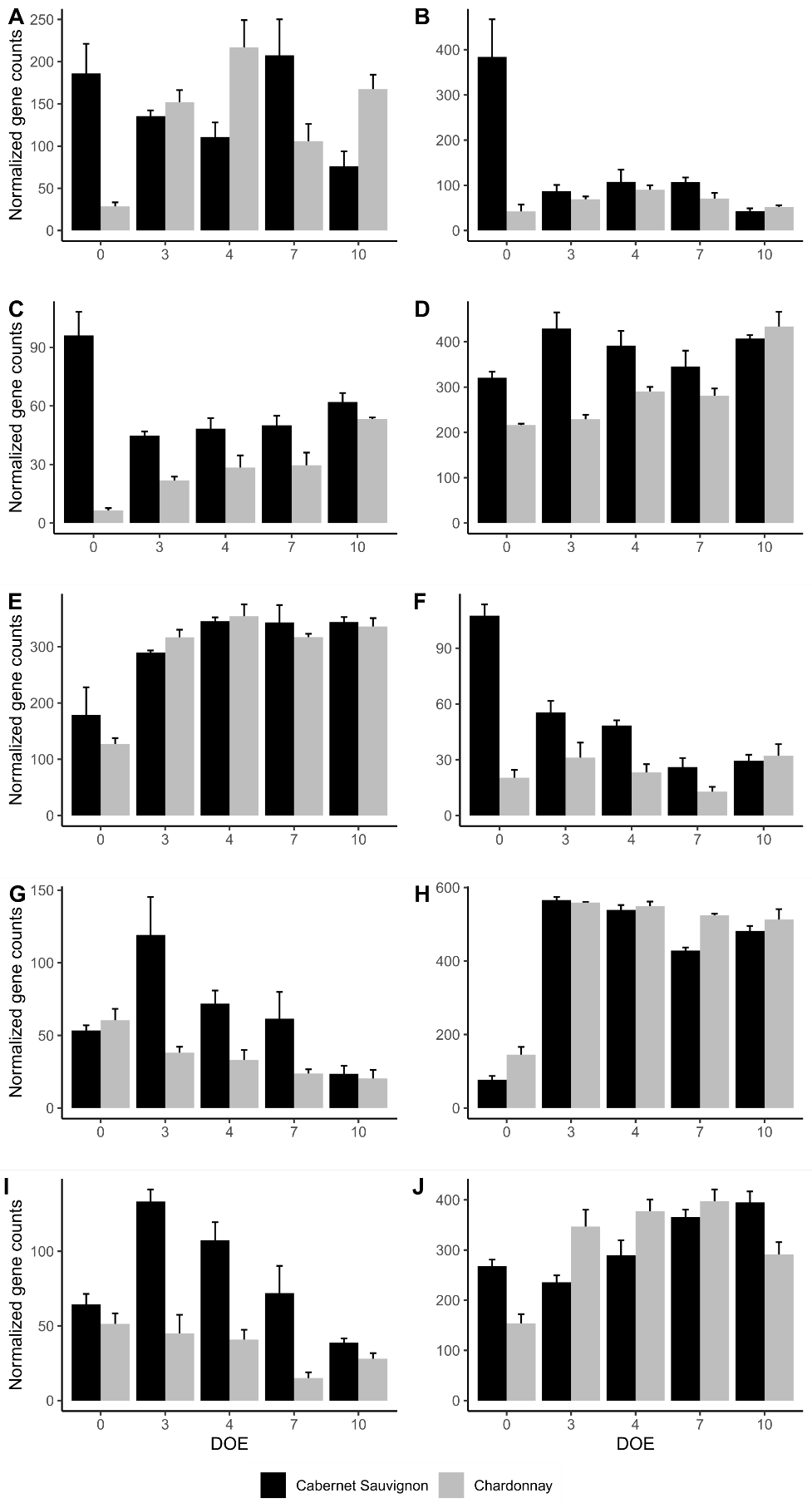


**Figure S4**. DESeq2 normalized gene counts of genes *VviNCED3* (**A**), *VviNCED4* (**B**), *VviNCED5* (**C**), *VviZEP1* (**D**), *VviZEP2* (**E**), *VviZEP3* (**F**), Absicic Aldehyde Oxidase (AAO) homologs (**G** to **I**) and *VviABA2* (**J**) in cv. Cabernet Sauvignon (black bars) and cv. Chardonnay (grey bars) throughout the experiment. DOE = day of experiment. Results are expressed as mean of 3 biological replicates ± standard error. *VviNCED3* = Vitvi05_01chr19g16940; *VviNCED4* = Vitvi05_01chr02g19450; *VviNCED5* = Vitvi05_01chr10g14230; *VviZEP1* = Vitvi05_01chr07g28260; *VviZEP2* = Vitvi05_01chr13g28160; *VviZEP3* = Vitvi05_01chr02g08870; *VviAAOs* = Vitvi05_01chr18g32800, Vitvi05_01chr06g13810, Vitvi05_01chr18g32770; *VviABA2* = Vitvi05_01chr13g23670.
